# The grand challenge in cellular neurophysiology

**DOI:** 10.3389/fncel.2026.1980835

**Published:** 2026-09-24

**Authors:** Björn Kampa, Arianna Maffei

**Affiliations:** 1 Department of Neurophysiology, Institute for Biology II, RWTH Aachen University, Aachen, Germany; 2 Department of Neurobiology and Behavior, Stony Brook University, Stony Brook, NY, United States

**Keywords:** glia, ion channels, neural circuits, neuron, neurophysiology, plasticity, synaptic receptors

## Introduction

The fascination with the brain as the seat of sensation and thought is long-standing. Descriptions of the effects of sedatives such as alcohol and opium and of the consequences of head injury date back to ancient Egypt (Krikorian, 1975; Sanchez and Burridge, 2007), and accounts of epilepsy to ancient Greece (Karamanou et al., 2017; Tsoucalas et al., 2017). Over the millennia many ideas about the anatomy and function of the brain were proposed, but the pivotal step toward our current understanding was the discovery of bioelectricity by Luigi Galvani in the late 1700s. Galvani's demonstration that animal tissue can generate electricity, and the debate it sparked with the physicist Alessandro Volta, drove the technological and conceptual advances that gave rise to cellular neurophysiology as we know it today (Piccolino, 1998; Verkhratsky and Parpura, 2014). Continued innovation and careful experimentation then established the properties of excitable membranes, the cellular organization of the brain, and the mechanisms of electrochemical signaling. This interplay between discovery and technology development continues, as every finding opens challenging new questions about brain function in health and disease. In this brief opinion we highlight the avenues of research in cellular neurophysiology that we, as editors, find particularly compelling.

## Cellular diversity and connectivity

Advances in anatomical, electrophysiological and molecular approaches have greatly expanded our ability to identify the diverse cell populations of brain tissue. Brain cells can be classified by morphology, location, electrophysiological properties, neurotransmitter identity, expression of specific proteins and full transcriptomic analysis (DeFelipe et al., 2013; Network, 2021; Chi et al., 2023; Gao et al., 2025; Jongbloets et al., 2026). In all species, from insects to humans, brain tissue comprises two major cell groups, neurons and glia. Neurons are broadly classified as excitatory or inhibitory according to the effect they exert on their post-synaptic targets, and are further distinguished by the transmitters they release and by other molecular markers they express. Glial cells show comparable diversity in morphology, molecular signature and function.

Important debates about what actually defines a cell type remain unresolved (Zeng, 2022). Detailed molecular profiling has dramatically increased the number of candidate cell types, sometimes yielding classifications that are inconsistent across research groups or analytical pipelines (Network, 2021; Yao et al., 2023). This increase in molecular resolution is powerful when paired with other criteria, as multimodal approaches such as Patch-seq illustrate by acquiring electrophysiology, morphology and transcriptome from the same neuron (Cadwell et al., 2016; Gouwens et al., 2020; Lee et al., 2023). Even so, the field still needs agreed strategies for mapping molecular, morphological and functional parameters onto one another, and a clearer criterion for what combination of parameters defines an actual cell type as opposed to a transient cellular state (Zeng, 2022; Gao et al., 2025). Mapping cell types across species is a related and equally open problem: broad categories such as glutamatergic and GABAergic neurons are well conserved, yet finer morphological and molecular divisions are hard to reconcile (Hodge et al., 2019; Berg et al., 2021). Spatial transcriptomics paired with functional recordings is a promising route toward resolving these questions.

A second line of open inquiry concerns how cell types interact. Are there connectivity modules that repeat across brain regions, or across areas, with comparable function in different species? Or are modules region-specific, shaped by the functional demands of a particular circuit? The possibility of canonical structures such as cortical columns or microcircuits is appealing, because it would reduce the complexity of neural networks to a manageable set of functional units. Evidence for such structures exists in several species (Mountcastle, 1997; Adams and Horton, 2009), but their functional implications remain debated (Martin and da Costa, 2010). Molecular approaches now allow neurons derived from a common precursor to be tracked (Brown et al., 2011; Li et al., 2012; Lin et al., 2023), testing whether shared origin biases connectivity and thereby contributes to module formation. In parallel, large-scale anatomical reconstruction (connectomics) is being used to chart circuit organization (Abbott et al., 2020; Celii et al., 2025; Ding et al., 2025; Dorkenwald et al., 2025; Gamlin et al., 2025; Weis et al., 2025). These approaches are technically demanding and depend on the development of new analytical tools and access to substantial computational power. As both advance, the identification of connectivity motifs within and across brain regions becomes a realistic goal, provided that structural motifs are validated against physiological measurements of what those connections actually do.

## Modes of communication

A fundamental aspect of neurophysiology is the study of excitable membranes and how their properties shape communication between brain cells. Great advances have been made in understanding neuronal excitability, synaptic transmission, electrical coupling and neuron–glia interactions. Genomic analyses have greatly expanded our view of the diversity of voltage-gated ion channels across species and cell types (Davila-Velderrain and Giese, 2025), while electrophysiological work has quantified the properties of distinct channels and receptor families and of individual members within them (Ranjan et al., 2011; Tripathy et al., 2017). This diversity underlies the characteristic excitability of neuronal and glial cell types. The subcellular location and clustering of specific channels can further confer specialized properties on particular compartments, such as the axon initial segment (Kerti et al., 2012; Nusser, 2012), or amplify synaptic inputs in distal dendrites (Park et al., 2025). Gap junctions can dramatically speed signal propagation between cells (Vaughn and Haas, 2022); they are restricted to certain cell types and are developmentally regulated (Montoro and Yuste, 2004; Vervaeke et al., 2012; Vaughn et al., 2024), adding a further dynamic dimension to circuit signaling.

Ligand-gated ion channels transform neurochemical signals into electrical currents. For each neurotransmitter several ionotropic receptors have been identified, differing in ligand affinity, activation and inactivation kinetics, and ionic permeability. Like voltage-gated channels, they are targeted to specific compartments and can sit pre- or post-synaptically, or outside synapses on dendritic shafts (Connolly et al., 1996; Brandon et al., 1999; Traynelis et al., 2010), with subunit composition strongly influencing their localization. Ligand-gated channels are also expressed by glia, where they shape the microenvironment around synapses and the activation state of specific glial populations (Lia et al., 2023). Despite these advances, much remains unknown about the relationship between the structure and function of membrane channels, and about the mechanisms controlling their expression, cell-type specificity, localization and activity-dependent regulation.

Communication in the brain is not confined to the synaptic cleft. Volume transmission, in which transmitters and neuromodulators diffuse to act on extrasynaptic and often high-affinity receptors, generates tonic currents and slow modulatory signals whose specificity derives from receptor distribution rather than from anatomical contact (Cavelier et al., 2005; Karadottir et al., 2005; Scimemi et al., 2006; Field et al., 2021). On a larger spatial scale, the movement of interstitial and cerebrospinal fluid distributes signaling molecules and clears metabolic waste (Iliff et al., 2012), and neurovascular coupling matches local blood flow and energy supply to activity through signaling among neurons, astrocytes, pericytes and endothelial cells (Attwell et al., 2010). Measuring these slow, spatially distributed processes with the resolution routinely achieved for synaptic events, and determining how they are integrated with fast transmission, is in our view one of the most important methodological challenges facing the field.

## Plasticity and flexibility of neuronal activity

Neural circuits must change with experience while remaining stable, and cellular neurophysiology is where this tension is best addressed. Hebbian mechanisms, from classical long-term potentiation and depression to spike-timing-dependent plasticity, remain the reference framework for associative learning (Magee and Grienberger, 2020). Yet the millisecond coincidence windows they require sit awkwardly with the seconds-long timescale of behavior. The description of behavioral timescale synaptic plasticity (BTSP) offered a compelling resolution: dendritic plateau potentials act as instructive signals that potentiate inputs active within several seconds, generating a hippocampal place field after a single trial (Bittner et al., 2017; Magee, 2026). BTSP raises questions we consider central for the coming years. Which molecular machinery reads out such long eligibility traces, and how is it gated by neuromodulation and inhibition? Does BTSP operate beyond CA1, in neocortical and subcortical circuits, and under which behavioral conditions do Hebbian and behavioral-timescale rules dominate? Answering these will require dendritic recording and imaging in behaving animals, combined with the ability to manipulate plateau potentials on demand.

Synaptic change is only one of several substrates. Neurons also adjust their intrinsic excitability by regulating channel expression and distribution (Debanne et al., 2019), remodel spines and axonal boutons (Bernardinelli et al., 2014b,a), and can switch the transmitter they release in response to sustained changes in activity (Li et al., 2020). Structural rearrangement is particularly intriguing, because the formation, stabilization and elimination of synaptic contacts unfold over hours to days and are therefore well matched to the persistence of memory (Lamprecht and LeDoux, 2004), yet the rules relating functional potentiation to lasting structural change are still poorly defined. Homeostatic mechanisms, including synaptic scaling and compensatory regulation of intrinsic properties, keep firing rates and excitation–inhibition balance within a functional range (Turrigiano and Nelson, 2004; Nelson and Turrigiano, 2008; Turrigiano, 2017; Wen and Turrigiano, 2024). How these mechanisms are coordinated in time and across compartments, whether they compete or cooperate, and how they are recruited by glia and by neuromodulatory systems, are open questions of exactly the kind this journal exists to publish.

## Cellular neurophysiology in health and disease

Many neurological and psychiatric conditions are, at their root, disorders of cellular excitability and synaptic function. Channelopathies provide the clearest illustration: loss of sodium channel function in GABAergic interneurons can produce circuit hyperexcitability and severe epilepsy (Yu et al., 2006), and the genotype-to-phenotype path now increasingly guides treatment choice. In neurodevelopmental disorders, altered synaptic maturation and disrupted excitation–inhibition balance during critical periods appear to be recurring themes, while in neurodegeneration synaptic and axonal dysfunction, along with network hyperexcitability, precede cell loss by years (Palop and Mucke, 2016). Reading these disorders as failures of specific cellular mechanisms, rather than as diffuse pathology, is what makes them tractable at the level of the single cell and synapse.

Progress here depends on model systems that combine physiological realism with experimental access, and studies using human tissue. Acute slices prepared from neurosurgically resected human cortex allow direct recording from human neurons and synapses, and have revealed properties without straightforward rodent equivalents, including distinctive dendritic excitability and integration in human layer 2/3 pyramidal cells (Ting et al., 2018; Gidon et al., 2020; Lee et al., 2023). Because such tissue is scarce and its viability limited, organotypic slice cultures (Stoppini et al., 1991) are a valuable complement. Human organotypic brain slice cultures preserve local circuitry for weeks and permit repeated imaging, genetic manipulation and chronic pharmacology (Schwarz et al., 2019). Stem-cell-derived neurons and brain organoids add scalable, patient-specific genetic backgrounds and are beginning to develop organized network activity (Trujillo et al., 2019), although their limited maturation and lack of full circuit architecture must be weighed carefully when interpreting physiological data. We see the systematic cross-validation of findings across non-mammalian and mammalian model systems and human slice, organotypic and stem-cell-based preparations as the central translational challenge. It is also the route by which cellular measurements become clinically useful: automated patch-clamp and multi-electrode platforms can now assign functional consequences to genetic variants at scale, turning a variant of uncertain significance into a gain- or loss-of-function classification that informs treatment.

## Outlook

The questions above share a common demand: measurements that link molecular identity, cellular physiology and circuit function within the same preparation, across species, and on timescales from milliseconds to weeks. Meeting it will require not only new technology but also transparent reporting, shared standards and datasets that others can reuse. Frontiers in Cellular Neurophysiology welcomes primary research, methods and review articles that take on these challenges, including negative and replication studies, and we look forward to the contributions of our community.
